# Supplementary material for: Integrating physiotherapy into primary care models: A scoping review protocol
Source: PLoS One. 2024 Dec 5;19(12):e0308023. doi: 10.1371/journal.pone.0308023 (PMC11620590; doi:10.1371/journal.pone.0308023)
Supplement: S2 Appendix — (DOCX) [file pone.0308023.s002.docx]

**Appendix 2: Search Strategy**

Ovid MEDLINE(R) ALL <November 07, 2024>

| **Search** | **Query** | **Results** |
| --- | --- | --- |
| 1 | **("Physical therap*" or "Physiotherap*")**.mp. *[mp=title, book title, abstract, original title, name of substance word, subject heading word, floating sub-heading word, keyword heading word, organism supplementary concept word, protocol supplementary concept word, rare disease supplementary concept word, unique identifier, synonyms, population supplementary concept word, anatomy supplementary concept word]* | 94385 |
| 2 | **("Primary health care" or "care, primary" or "care, primary health" or "healthcare, primary" or "primary care" or "primary care model" or "primary medical care" or "patient care team" or "family health team" or "models of care").**mp. *[mp=title, book title, abstract, original title, name of substance word, subject heading word, floating sub-heading word, keyword heading word, organism supplementary concept word, protocol supplementary concept word, rare disease supplementary concept word, unique identifier, synonyms, population supplementary concept word, anatomy supplementary concept word]* | 281295 |
| 3 | **("patient's medical home" or "primary assessor" or "advanced practice" or "first contact" or "direct access" or "rapid access" or "triage" or "team-based").**mp. *[mp=title, book title, abstract, original title, name of substance word, subject heading word, floating sub-heading word, keyword heading word, organism supplementary concept word, protocol supplementary concept word, rare disease supplementary concept word, unique identifier, synonyms, population supplementary concept word, anatomy supplementary concept word]* | 59789 |
| 4 | 2 or 3 | 334006 |
| 5 | 1 and 4 | 4989 |
| 6 | limit 5 to yr="2003-Current" | 4078 |
